# The Critical Role of Pulse Oximetry Screening for Congenital Heart Disease and Patent Ductus Arteriosus in Newborns

**DOI:** 10.7759/cureus.75388

**Published:** 2024-12-09

**Authors:** Ioana Rosca, Daniela-Eugenia Popescu, Ana Maria Cristina Jura, Alina Turenschi, Cristina Filip, Alexandru Dinulescu, Andreea T Constantin

**Affiliations:** 1 Neonatology, University of Medicine and Pharmacy Carol Davila, Bucharest, ROU; 2 Neonatology, Clinic Hospital of Obstetrics and Gynecology Prof. Dr. Panait Sirbu, Bucharest, ROU; 3 Obstetrics-Gynecology and Neonatology, Victor Babeş University of Medicine and Pharmacy, Timisoara, ROU; 4 Pediatrics, Ploiești Pediatric Hospital, Ploiești, ROU; 5 Pediatric Cardiology, Marie Curie Children's Hospital, Bucharest, ROU; 6 Pediatrics, Carol Davila University of Medicine and Pharmacy, Bucharest, ROU; 7 Pediatrics, Alessandrescu-Rusescu National Institute of Mother and Child Health, Bucharest, ROU

**Keywords:** congenital heart disease, neonatal intensive care unit, neonatology, new diagnostic approach in congenital heart disease, parent support, pediatric cardiology, prenatal screening, pulse oximetry

## Abstract

Introduction. Congenital heart disease (CHD) is diagnosed with high prevalence. Pulse oximetry and clinical examination are screening tools to aid in obtaining a CHD diagnosis.

Materials and Methods. We conducted a retrospective longitudinal study over three years, screening 1188 newborns admitted to the neonatal intensive care unit (NICU) during the first 72 hours of life.

Results. Two hundred one newborns were diagnosed with CHD. Almost all (n=198) had a positive screening test during the 72 hours, while three had a negative screening test but were later diagnosed using echocardiography. The prevalence rate of CHD was 16.67%. A confusion matrix was constructed with 198 true positives (TP), 3 false negatives (FN), no false positives (FP), and 987 true negatives (TN). The sensitivity of the screening test was 98.51%, while the specificity was 100%, with no false positives. In our study, a total of nine deaths occurred due to CHD or complications of CHD. Using the Kaplan-Meier method, the survival probability appeared to be slightly above 0.9 (95.5%), indicating that almost all patients survived past 72 hours after being diagnosed with CHD.

Conclusion. Pulse oximetry is an effective, low-cost, and easy-to-use method that is useful for assessing and diagnosing CHD.

## Introduction

Congenital heart disease (CHD) is a congenital disorder diagnosed with high prevalence, affecting approximately 0.8% to 1.2% of all live births [1]. CHD encompasses a comprehensive range of abnormalities manifesting at birth, including numerous defects that may occur individually or in conjunction. The atypical configuration of the cardiac chambers, valves, or major blood vessels in individuals diagnosed with CHD disrupts the typical hemodynamic pattern [2]. While some defects are mild and may not require intervention, others are severe and need treatment shortly after birth [3].

Although there are now more clearly defined risk factors, the cause of most CHDs is still unknown. A well-established causal relationship can only be defined in 10 to 15% of cases [4]. The majority of adverse pregnancy outcomes can be attributed to genetic-environment interactions. This thorough examination of risk factors and responses to congenital heart defects reveals a complex network of functional interactions between genomic variations and environmental exposures. These interactions play a crucial role in regulating essential biological systems during the heart's development [5]. Studies often highlight risk factors for CHD that are linked to extreme maternal and paternal ages, particularly those under 21 years and over 35 years of age, as well as unfavorable socioeconomic status. In addition, maternal health status plays a crucial role, and diseases such as diabetes with insulin use, autoimmune pathology such as lupus, hypertension, obesity, and unspecified febrile illness contribute [6]. Specific infectious diseases such as rubella and parvovirus, smoking, and gestational stress are also included. Moreover, there is a correlation between maternal and paternal exposure to alcohol and other substances and CHD [7].

Over the past decades, mortality for this category of patients has decreased significantly [8]. Nevertheless, the mortality rate of CHD exhibits important global variability. In countries with limited access to healthcare services, the mortality rates are higher compared to developed nations [9].

Research has demonstrated that the utilization of pulse oximetry screening (POS) in neonates can effectively improve the identification of critical congenital heart disease (CCHD) [10]. Critical congenital heart disease has been characterized in the literature as duct-dependent CHD that poses a significant risk to life if left untreated during the neonatal period. These conditions include duct-dependent pulmonary and systemic anomalies. Pulmonary anomalies include pulmonary atresia with an intact ventricular septum, pulmonary stenosis, tetralogy of Fallot, total anomalous pulmonary venous return, transposition of the great arteries (TGA), tricuspid atresia, and truncus arteriosus. Duct-dependent systemic abnormalities include coarctation of the aorta, interrupted aortic arch, hypoplastic left heart syndrome, and aortic stenosis [11]. Pulse oximetry is an easily accessible screening method, cost-efficient, and a non-invasive tool. It remains an underutilized screening method in newborns, although it can be easily performed by nurses or doctors at a patient's bedside [12]. Furthermore, postnatal detection by pulse oximetry combined with clinical assessment are useful methods for CHD screening in most areas. Clinical assessment includes standard physical examination (observation, palpation, and auscultation), chest radiograph, and electro- and echocardiography [13].

Our study aimed to use pulse oximetry as a rapid screening tool for prompt diagnosis of CHD in newborns. This approach aimed to quickly and accurately identify CHD, which poses significant risks if undetected and untreated early. By implementing pulse oximetry within the first 72 hours of life, we sought to enhance early detection rates of CHD, enabling timely medical interventions and improving and affecting an infant's survival and health outcomes. Our purpose was to detect anomalies through pulse oximetry and take necessary steps according to the condition's severity grade. This screening was designed to raise awareness among physicians about its importance, emphasizing that they should use this diagnostic tool to confirm or refute potential CHD diagnoses.

This article was previously posted to the PrePrints.org server on April 7th, 2024; DOI: 10.20944/preprints202404.0470.v1.

## Materials and methods

We conducted a longitudinal retrospective study to evaluate the prevalence of CHD in a tertiary referral center in Bucharest, Romania. The study focused on infants born with complex cardiovascular malformations admitted to our clinic over three years (January 2021 - December 2023). Additionally, we assessed the effectiveness of screening methods in identifying patients with CHD and their survival rates. During the abovementioned period, 11,013 patients were born in our clinic, of which 1188 newborns were admitted to the neonatal intensive care unit (NICU) and were included in the study.

The inclusion criteria represented patients born in our clinic and admitted to the NICU, antenatal suspicion of CHD (including maternal history, fetal echocardiography, and other clinical indicators), and the need for pulse oximetry monitoring. Those with co-existing medical conditions, such as congenital anomalies not related to congenital disease, and those born in other maternities and transferred into our clinic were excluded.

All preterm patients are admitted to our NICU for monitoring. Additionally, those born from high-risk maternal pathology (described in the results section) further justified the need for NICU admission to ensure comprehensive care and early intervention for the infants. Moreover, patients with signs of respiratory distress syndrome, low birth weight, or suspicion of CHD demanded immediate and detailed evaluation, which consequently led to access to the NICU.

Ethical approval was obtained from the Research Ethics Committee of the Clinical Hospital of Obstetrics and Gynecology" Prof. Dr. P. Sârbu", Bucharest, no.10038/01.08.2024.

Screening methods for CHD detection included pulse oximetry in both preductal and postductal locations (right hand for preductal measurements and either foot or left hand for postductal measurements, respectively) and clinical assessment (observation, auscultation). All positive screening tests required an echocardiographic exam, which led to timely confirmation or disconfirmation. Pulse oximetry and clinical assessment were performed by a trained neonatologist, and cardiac ultrasonography was performed by a cardiologist specialized in pediatric cardiology. A single positive screening test was used as a trigger for echocardiographic confirmation to reduce the number of unnecessary echocardiograms. While this approach may lead to some false-positive results, we believe that the potential benefits of early diagnosis outweigh the risks of overdiagnosis.

A positive pulse oximetry screen (POS) was determined by a SpO_2_ level below 90% in the right hand, or a SpO_2_ level between 90% and 94% in either location (preductal or postductal location), or a difference of more than 3% between the two locations (measured twice with a one-hour interval). A negative pulse oximetry screen was determined by an oxygen saturation (SpO_2_) level of 95% or higher in the right hand, with a difference of 3% or less between the preductal and postductal measurements.

The statistical analysis used an array of essential metrics such as sensitivity, specificity, positive predictive value, prevalence, and likelihood ratio, which were employed to evaluate the accuracy of the screening method. Furthermore, the Kaplan-Meier function was applied to assess the survival rates of patients diagnosed with CHD, offering valuable insights into their prognosis. It is worth noting that all statistical analysis was performed using R Studio software (Version 2023.09.1+494 for Mac). The following libraries were used: "tidyverse", "pROC", "survival", "epiR".

## Results

During the studied period (January 2021 - December 2023), 11,013 patients were delivered to our clinic. 948 (79.7%) neonates were born from personalized follow-up pregnancies (PPUP), while 192 (16.16%) were partially followed-up, and 48 (4.13%) pregnant women did not go to the obstetrician up until admission for labor and birth. Throughout the analyzed timeframe, less than a quarter of newborns required admission to the NICU (1188 patients, 10.7%). Of them, 1102 (92.7%) were preterm, and 86 (7.3%) were term neonates.

Maternal pathology included gestational diabetes (n=17), SARS-CoV-2 infection (n=6), placenta praevia (n=21), neuromuscular pathology (n=1), obesity (n=35), high arterial blood pressure (n=16), genito-urinary infections (n=31), anemia (n=54), cerclage (n=11), and hypothyroidism (n=9). These characteristics underscore the complex medical needs and critical conditions managed within the NICU, highlighting the need for monitoring and special care for these newborns.

Pulse oximetry screening (both pre-and postductal screening) and clinical examination on admission and up to 72 hours of life were performed for all patients admitted to the NICU. Although 23 patients had a prenatal diagnosis of CHD, we included them in the initial POS as well.

Clinical examination consisted of identification of cyanosis, SpO_2_<95% or major clinical signs of cardiorespiratory distress, systolic murmur on auscultation, or cardiogenic shock (for complex CHDs). In our study, out of 201 patients diagnosed with CHD, 175 exhibited clinical signs indicative of this condition. The most common clinical manifestations included cyanosis, detected in 28 patients (13.93%), a systolic murmur observed in 133 patients (66.17%), and cardiogenic shock, particularly in those with complex CHDs, seen in 14 patients (6.97%).

Over the three years, 201 newborns were diagnosed with CHD. A great number (n=198, 98.5%) had a positive screening test during the 72 hours, while three had a negative screening test and were diagnosed using echocardiography. Patient characteristics are described in Table 1.

**Table 1:** Characteristics of the study group Notice the high percentage of systolic murmurs identified on clinical examination in our patients. GA: Gestational age; SARS-CoV-2: Severe acute respiratory syndrome coronavirus 2; CHD: Congenital heart disease

Variables	Patients (n=201)
Gender	
Male	110
Female	91
Pregnancy	
Natural Conception	173
In vitro fertilization	28
Orotracheal intubation	58
Underwent surgery	16
Deaths	9
Gestational age	
Term newborns (>37 weeks GA*)	64
Preterm newborns (<37 weeks GA)	137
Late preterm (34 – 37 weeks GA)	50
Low birthweight (<2500g)	31
Very low birth weight (<1500g)	45
Extremely low birth weight (<1000g)	11
Maternal pathology	
Gestational diabetes	17
SARS-CoV-2 infection	6
Placenta praevia	21
Neuromuscular pathology	1
Obesity	35
High arterial blood pressure	16
Genito-urinary infections	31
Anemia	54
Cerclage	11
Hypothyroidism	9
Prenatal CHD diagnosis	23
Clinical examination	
Cyanosis, SpO_2_<95% or major clinical signs of cardiorespiratory distress	28
Systolic murmur	133
Cardiogenic shock (complex CHDs)	14

The prevalence rate of CHD in the screened intensive care unit population was approximately 16.67%. A confusion matrix was constructed with 198 true positives (TP), three false negatives (FN), no false positives (FP), and 987 true negatives (TN). The sensitivity of the screening test (the ability to correctly identify those with CHD) is about 98.51%, while the specificity (the ability to correctly identify those without CHD) is 100%, as there are no false positives.

The likelihood ratio was evaluated as well. For our study, the positive likelihood ratio (LR+) is infinite. In practice, this suggests that a positive screening result is a strong indicator of the disease, given there are no false positives. However, the Negative Likelihood Ratio (LR-) is approximately 0.015, indicating that a negative screening result moderately decreases the likelihood of CHD.

The high sensitivity and specificity values of the screening test demonstrate its effectiveness in accurately identifying individuals with and without CHD, and with no false positives reported, the test's reliability is commendable. The infinite LR+ underscores the strong predictive value of a positive screening result. Conversely, the low LR- suggests that a negative result can moderately reduce the probability of CHD. These results highlight the importance of using this screening test as a valuable tool in assessing CHD risk and making informed healthcare decisions.

**Figure 1:**
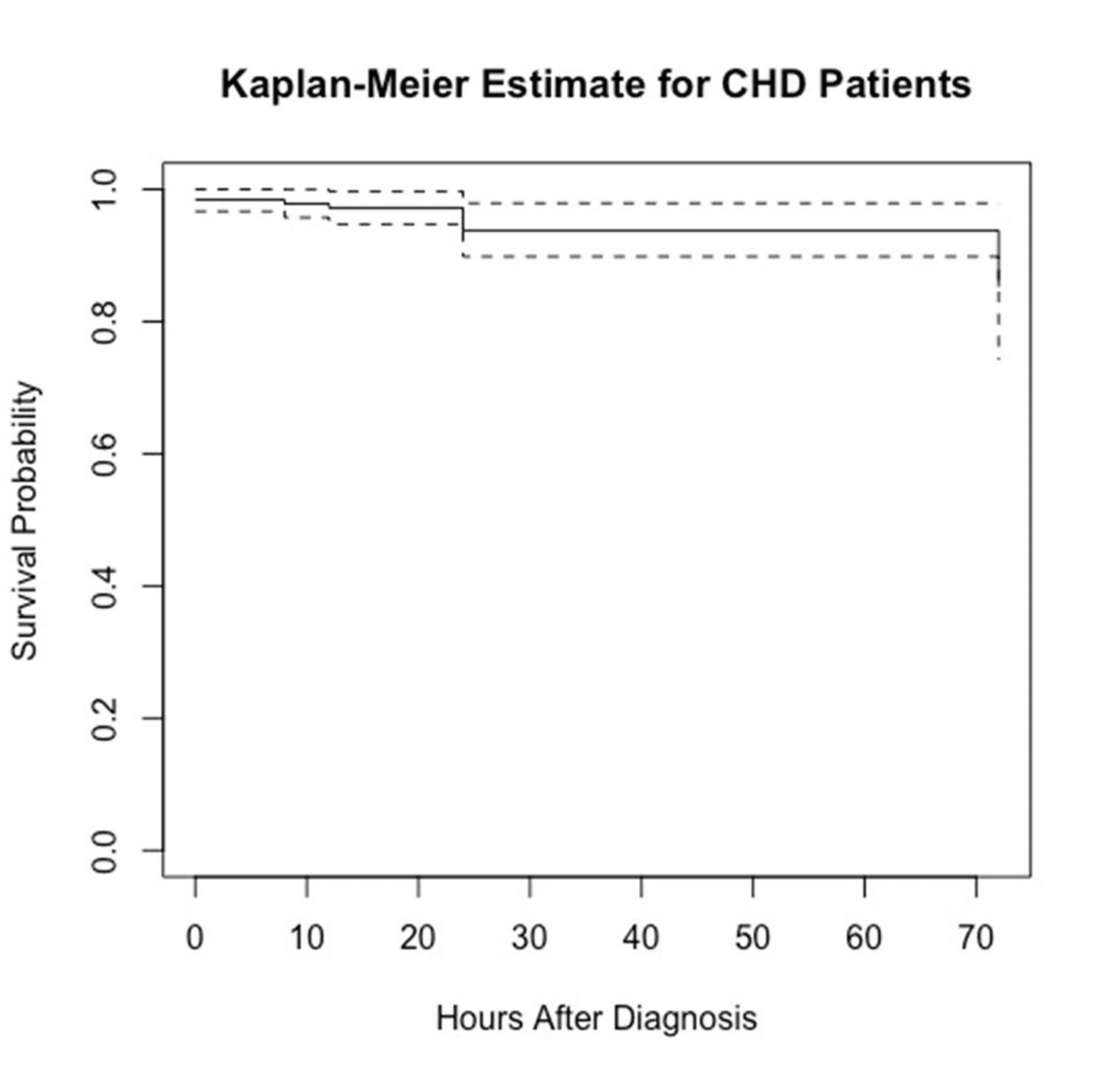
Kaplan-Meier survival analysis up to 72 hours for patients with congenital heart disease

The Kaplan-Meier estimate is a widely employed statistical method for comprehending the survival distribution of a population. It is frequently utilized in clinical research to estimate the probabilities of a patient's survival over a given period. In our study, a total of nine deaths occurred. All patients began with a survival probability of 1 (100%) at the time of diagnosis (0 hours). The Kaplan-Meier graph (Figure 1) for CHD illustrates the survival probability over time, measured hours after diagnosis. There is a noticeable decrease in the survival probability within the first 12 hours, reflecting three deaths. Another decline is observed at 24 hours, corresponding to an additional four deaths, further reducing the survival probability. The final decrease occurs by 72 hours, with two more deaths accounted for, further reducing the survival probability. Between these drops, the survival probability remains constant, indicating periods where no deaths were recorded. The dashed lines after the last drop at 72 hours suggest the presence of censored data. Censored data indicates that some patients did not experience the event (death) by the end of the observation period or were lost to follow-up.

By the end of the study period (72 hours), the survival probability appears to be slightly above 0.9 (95.5%), indicating that approximately 90% of the patients survived past 72 hours after being diagnosed with CHD. The Kaplan-Meier graph provides a detailed and insightful visualization of the survival probabilities for CHD patients following their diagnosis. It clearly illustrates the impact of time on survival rates and highlights the critical periods where a decline in survival probability occurs due to patient deaths. The presence of censored data is also acknowledged, indicating that not all patients experienced the event (death) within the observation period. Overall, by analyzing this data, researchers can gain valuable insights into the progression of CHD and better understand the factors influencing patient outcomes over time.

The POS test and clinical examination were followed by an echocardiography for all patients, which a skilled pediatric cardiologist performed. All diagnoses can be observed in Table 2. PDA represented the most frequent anomaly. While 19 (16.9%) required pharmacological closure, most of them (n=93, 83.1%) did not require either medication or surgical correction. Although PDA in neonates is not typically considered a pathological finding, we included this diagnosis in our study because it requires attention, especially in preterm infants. PDA can worsen respiratory distress syndrome and impair oxygenation, causing significant clinical complications. Identifying and monitoring PDA allows healthcare providers to manage these cases cautiously, ensuring timely interventions to reduce potential adverse effects. Including PDA in our study emphasizes the importance of comprehensive neonatal screening and vigilant management to improve outcomes for these vulnerable patients.

Almost a quarter of patients were diagnosed with ventricular septal defect (VSD) (n=34, 16.9%), and a similar percentage with pulmonary stenosis (n=27, 13.4%). Several cases of transposition of the great vessels, coarctation of the aorta, double aortic arch, tetralogy of Fallot, bicuspid aortic valve, hypoplastic left heart syndrome, or pulmonary valve atresia were also found. In all cases, VSD was associated with PDA, which led to an earlier diagnosis.

**Table 2:** Congenital heart diseases diagnosed in the study.

Diagnosis	Patients (n=201)
Ventricular septal defect	34 (16.91%)
Patent ductus arteriosus	112 (55.72%)
Transposition of the great vessels	2 (0.99%)
Coarctation of the aorta	8 (3.98%)
Double aortic arch	1 (0.49%)
Pulmonary stenosis	27 (13.43%)
Grade 5 mitral regurgitation; dysplastic mitral valve	2 (0.99%)
Tetralogy of Fallot	4 (1.99%)
Bicuspid aortic valve	2 (0.99%)
Pulmonary valve atresia	1 (0.49%)
Tricuspid valve atresia	1 (0.49%)
Hypoplastic left heart syndrome	6 (2.98%)
Hypoplastic aortic arch	1 (0.49%)

**Figure 2:**
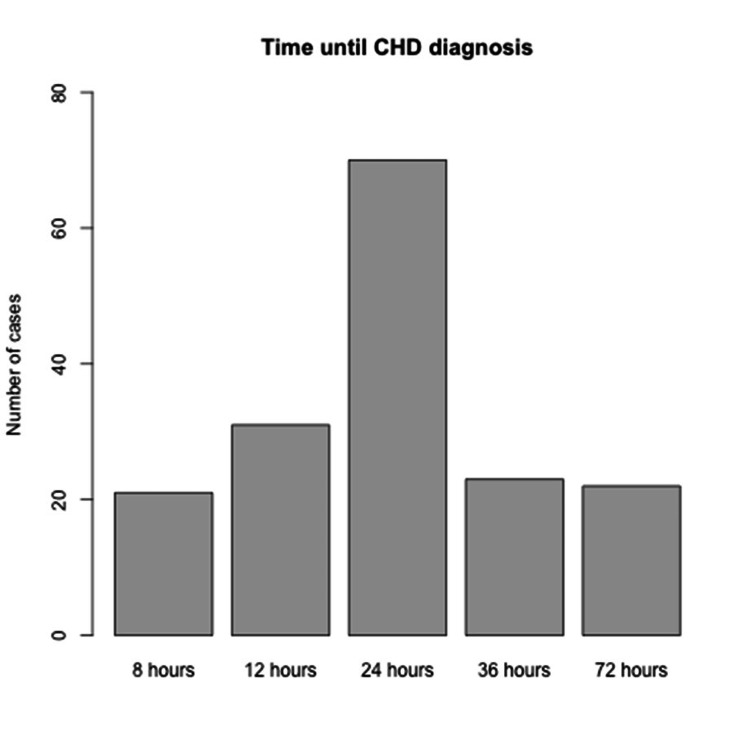
Number of cases/time (hours) from birth to diagnosis Twenty-one cases were diagnosed by 8 hours of life, 31 by 12 hours, 70 by 24 hours, 23 by 36 hours, and 22 more by 72 hours of life. CHD: Congenital heart disease

The timeline between the screening test and diagnosis, as vividly illustrated in Figure 2, reveals a crucial pattern in the early detection of this condition. Strikingly, a substantial majority, over half of the cases, were successfully identified within a mere 72 hours after birth, totaling an impressive 122 diagnoses. This rapid identification rate underscores the efficiency and effectiveness of our current diagnostic protocols, demonstrating their potential to dramatically improve patient outcomes through early intervention.

It's important to note that this graph intentionally excludes the 23 prenatally diagnosed cases. This deliberate omission highlights and emphasizes the remarkable success of our postnatal diagnostic procedures. By focusing solely on postnatal diagnoses, we can more accurately assess and appreciate the speed and precision with which our medical teams can identify and respond to this condition in newborns.

**Figure 3:**
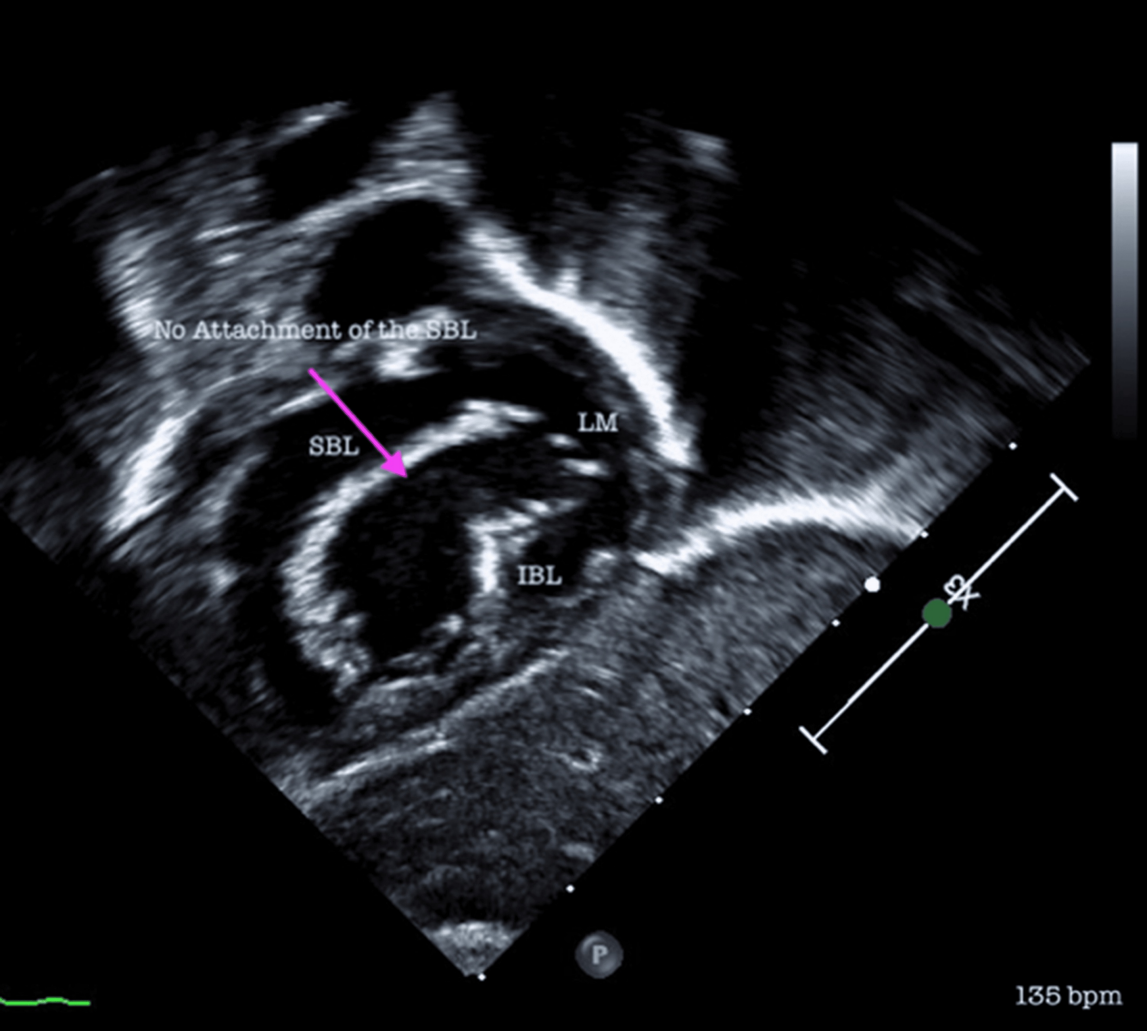
Ventricular septal defect Rastelli TYPE C – no superior leaflet bridging attachment. A subcostal view in transthoracic echocardiography shows a common atrio-ventricular valve with five leaflets and no attachment of the superior bridging leaflet (Atrioventricular septal defect Rastelli type C). SBL: Superior bridging leaflet; LM: Left mural leaflet; IBL: Inferior bridging leaflet

**Figure 4:**
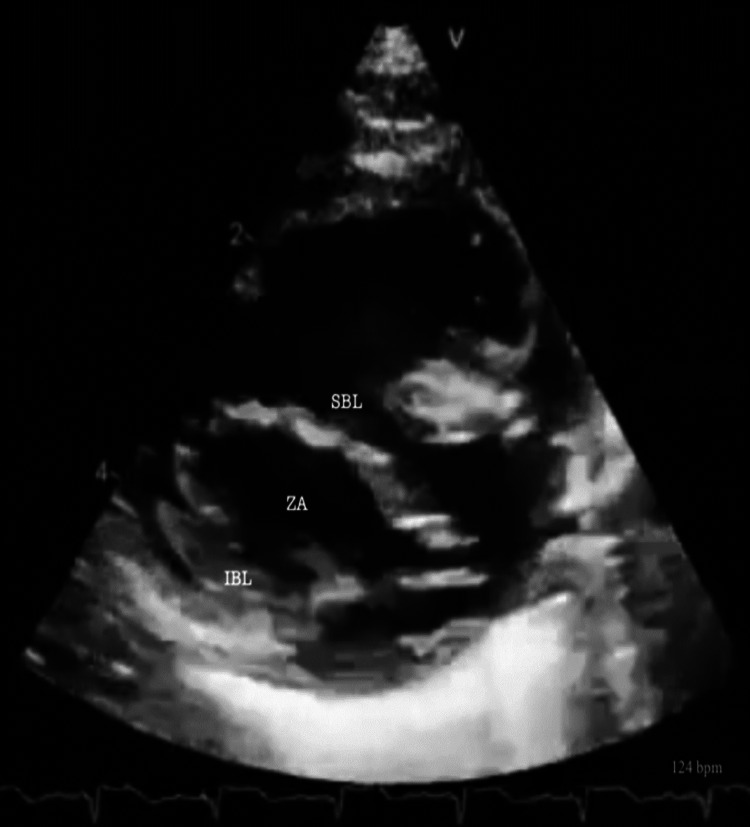
Ventricular septal defect Rastelli TYPE C – no superior leaflet bridging attachment The modified parasternal short axis shows a complete atrioventricular septal defect with no attachment to the superior bridging leaflet. SBL: Superior bridging leaflet; ZA: Zone of apposition; IBL: Inferior bridging leaflet

**Figure 5:**
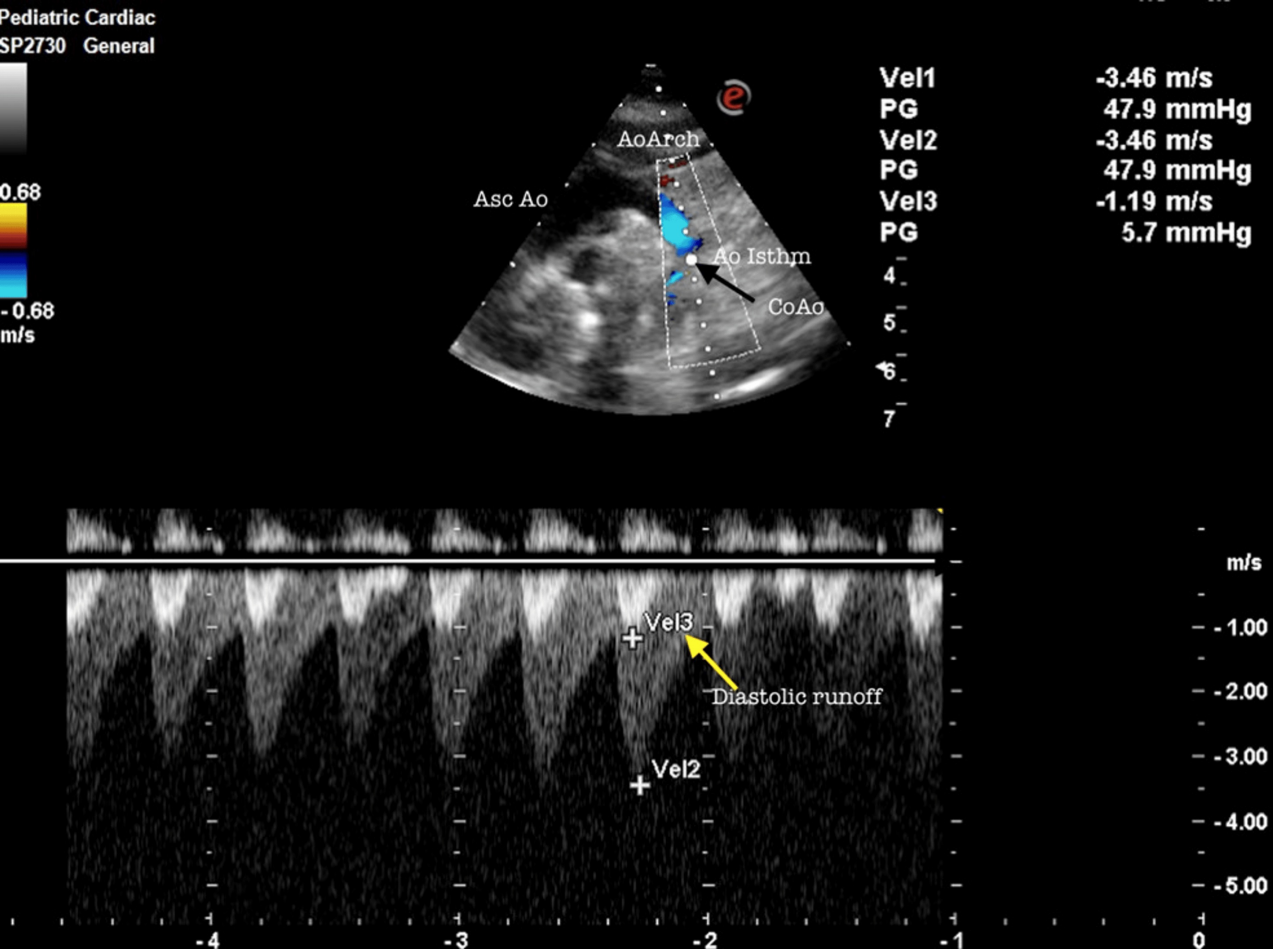
Coarctation of the aorta Suprasternal view in transthoracic echocardiography showing flow velocity acceleration with diastolic runoff (yellow arrows) in the distal 5th aortic arch. AscAo: Ascending aorta; AoArch, aortic arch; Ao isthm: Aortic isthmus; CoAo: Coarctation of the aorta

**Figure 6:**
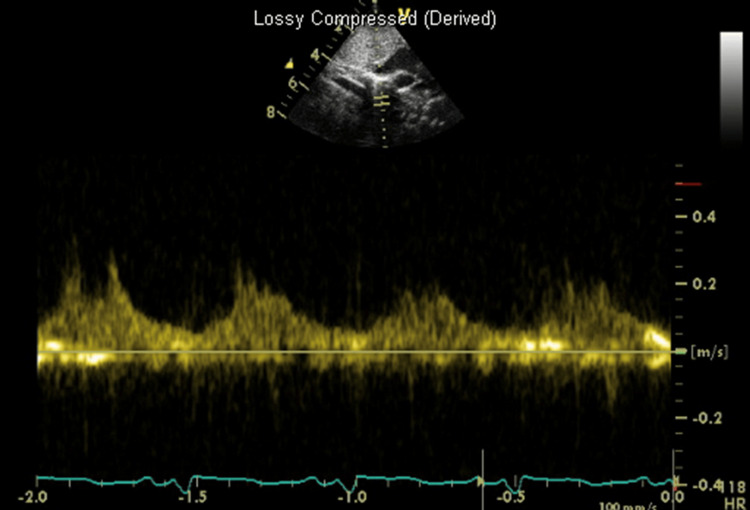
Coarctation of the aorta Subcostal view in transthoracic echocardiography showing reduced flow velocity at the abdominal aorta level with a diastolic “tail” consistent with severe coarctation of aorta with reduced pulse width.

**Figure 7:**
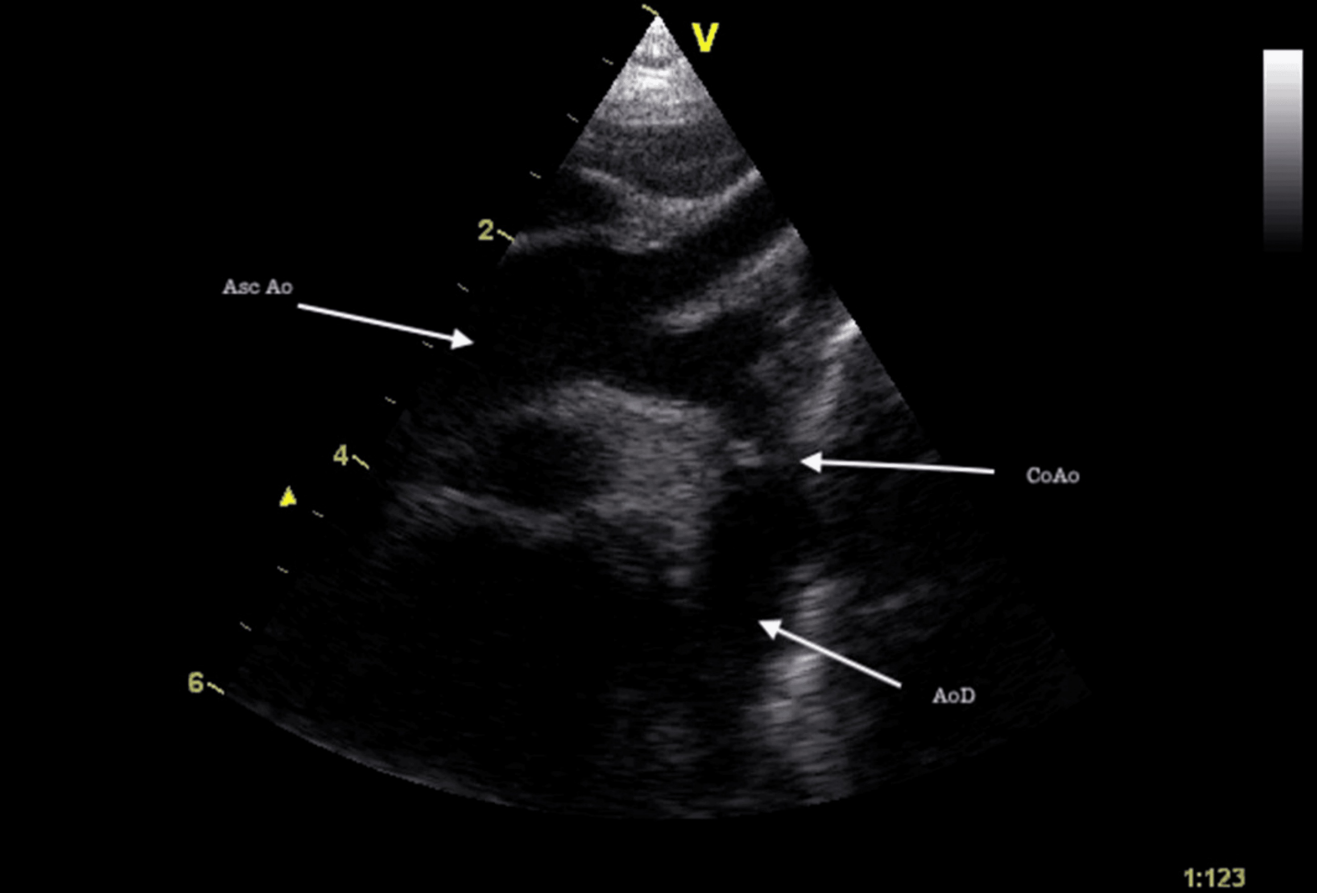
Coarctation of the aorta A suprasternal long-axis view showing significant preductal coarctation. There is a caliber discrepancy between the Asc Ao and the transverse arch. Asc Ao: Ascending aorta; CoAo: Coarctation of aorta; AoD: descending aorta.

A small percentage (n=16, 7.9%) required transfer and surgical correction of the identified CHD. All 16 of themunderwent surgery in our country (Bucharest, Cluj-Napoca, and Târgu-Mureș). Several diseases can be observed in the echocardiographic exams shown in Figures 3-7.

## Discussion

This study focused on implementing a screening program for CHD in newborns and subsequent monitoring of early-life patient outcomes. Future research should prioritize longitudinal follow-up to understand long-term outcomes beyond the initial 72-hour window. Detailed investigation into the causes of mortality and critical periods for intervention could offer insights for improving care. The ultimate goal is to enhance early detection and treatment to improve survival rates and quality of life for infants with CHD.

During the three-year study period (January 2021- December 2023), 11,013 newborns were born into our clinic, and 1188 were admitted to the NICU. All of these patients were screened using pulse oximetry and clinical assessment for 72 hours, and 201 were diagnosed with CHD (198 had a positive screening test, and 3 had a negative screening test but were later diagnosed with CHD). A growing body of literature presents evidence supporting the effectiveness of pulse oximetry as a screening test for CCHD in newborns [14].

The implementation of CCHD screening during the first hours of life in tertiary maternities has shown promising results. In our study, POS during the first 72 hours of life has proven a sensitivity of 98.51%. Gopalakrishnan S. et al. report a 75% sensitivity and 99.29% specificity, with a false positive rate of 0.81% [11], while Singh et al. describe a higher sensitivity (85.7%) and 99.3% specificity [15].

The occurrence of CHD in newborns is significantly influenced by maternal health history and parental family history. Maternal medical conditions, such as gestational diabetes, hypertension, and obesity, are recognized to heighten the risk of CHD by impacting fetal development. For instance, gestational diabetes can result in elevated blood glucose levels during crucial periods, whereas hypertension and obesity can impact blood circulation and nutrient supply to the developing fetus. Autoimmune diseases, such as lupus, can also have an adverse effect on the development of the fetal heart. In addition, maternal infections during pregnancy, such as rubella, and exposure to harmful substances like alcohol and tobacco increases the risk of CHD. Both extremely young and older maternal age, as well as low socioeconomic status, are linked to a higher likelihood of congenital anomalies [16]. This could be because of limited access to prenatal care and increased exposure to harmful substances. The parental family history is also a significant factor, as genetic predispositions can raise the chances of offspring developing CHD. Genetic mutations or syndromes that are passed down from parents, such as Down syndrome, frequently exhibit a greater occurrence of heart defects. Coronary heart disease often occurs due to multifactorial inheritance, which involves the combination of genetic and environmental factors that collectively raise the risk. Gaining knowledge about the medical backgrounds of one's family and mother can provide valuable information for identifying potential health issues at an early stage, implementing preventive measures, and offering genetic counseling. The authors ought to examine the interaction between genetics and environment, the significance of preventive measures, and the consequences for research and clinical practice. Additional avenues for mitigating the risk of CHD could involve the examination of public health strategies, including the promotion of maternal health education and the enhancement of prenatal care accessibility. By recognizing these factors, healthcare providers can more effectively evaluate risks and implement strategies to enhance outcomes for affected families [16].

Hu X. et al. underlined the importance of POS in the NICU, but additionally, the significance of both pre- and postductal monitoring of saturations. The authors highlighted the fact that without simultaneous monitoring of SpO_2_, the screen for CCHD would have failed to identify one case of transposition of the great vessels and six cases of persistent pulmonary hypertension, with delayed treatment and grave consequences [17]. Likewise, our screening consisted of preductal and postductal saturation 72 hours assessment, leading to early detection and echocardiographic diagnosis. 

Ma X. et al. conducted a 5-year screening program for detecting CHD using pulse oximetry and cardiac murmur auscultation for 801,831 newborns from 6 to 72 hours of life. High sensitivity and specificity of the cardiac murmur auscultation method in clinical practice were observed for both critical (100.00% and 97.72%) and major CHD (98.47% and 97.76%) [18]. In our study, only 133 had a systolic heart murmur (66.1%), but they had a POS screening test.

A pediatric cardiologist plays a major role in confirming a diagnosis of CHD. Nevertheless, neonatologists can detect structural CHDs using cardiac ultrasonography. The goal is to involve a pediatric cardiologist for a precise and early diagnosis. On the other hand, if a scan shows a heart that seems to be structurally normal, it could decrease the number of referrals to pediatric cardiology and potentially prevent the need for patient transfers [19].

Implementing oximetry to the standard physical examination aids in identifying a greater number of newborns with critical congenital heart defects without significantly raising the rate of false-positive results. This discovery has the potential to decrease the morbidity and mortality rates linked to discharging newborns from the hospital without a timely diagnosis. This leads to a more rapid diagnosis and transfer for surgical correction and better outcomes [20].

A key component of the treatment approach is promptly detecting CCHD. This includes prenatal diagnosis. In our study, it was present only for 23 out of 201 cases, as only 79.7% of newborns came from correctly followed-up pregnancies. Prenatal diagnosis and newborn screening are two methods used to detect these conditions at an earlier stage. Prenatal diagnosis is particularly useful as it allows for careful planning of management strategies in collaboration with family and care providers [21].

Pulse oximetry represents a relatively low-cost, simple to use and effective method for CCHD screening. However, combining pulse oximetry with clinical assessment is a viable approach worth considering for long-term implementation. Enhancing the accessibility of treatment is essential for maximizing the potential health advantages of screening [22]. Pulse oximetry is recommended for screening CHD in newborns because it is non-invasive, cost-effective, provides quick results, and is easy to use. Although it cannot substitute more comprehensive diagnostic tests such as echocardiography, it plays a crucial role as an initial screening method to identify newborns who are at risk for CHD. This helps ensure that these infants are promptly referred for further evaluation and treatment. By incorporating pulse oximetry into regular neonatal care, medical professionals can greatly enhance the early identification and prognosis of infants with CCHD.

Including PDA in the CHD diagnosis is crucial due to its significant impact on neonatal health, especially in preterm infants. Although PDA is not a pathologic finding, it can exacerbate conditions such as respiratory distress syndrome and impair oxygenation, which are critical concerns in preterm neonates. Identifying and continuously monitoring a known PDA allows healthcare providers to implement timely interventions and manage the condition more effectively. This proactive approach can mitigate potential complications, stabilize the infant's respiratory status, and improve overall outcomes.

Echocardiography remains the most definitive and useful diagnostic tool for CHD, providing detailed anatomical and functional information essential for accurate diagnosis and management. However, integrating screening tests, such as pulse oximetry, allows for earlier detection of potential CHD cases. These screening tests can identify newborns at risk for CHD within the critical first 72 hours of life, facilitating prompt referral for echocardiographic evaluation. Early screening thus enhances the timeliness of diagnosis, enabling healthcare providers to initiate necessary interventions sooner, which can significantly improve clinical outcomes and reduce morbidity and mortality associated with CHD in newborns.

The primary objective of our comprehensive study was to showcase the remarkable impact of integrating POS, clinical assessment, and cardiac ultrasonographic examination in enhancing the early detection rates of CHD within the crucial 72-hour window following birth. This approach facilitates swifter interventions and significantly improves neonatal outcomes, thereby reducing hospital NICU stays, mitigating morbidity rates, and ultimately lowering mortality risks within the vulnerable neonatal cohort.

Although the present study emphasizes the reliability and effectiveness of pulse oximetry as a screening tool in the NICU, further research is required to assess its performance in comparison to other diagnostic methods such as echocardiography, physical examinations, and electrocardiogram (ECG). Multivariate analysis can enhance comprehension of the strengths and limitations of pulse oximetry, specifically in detecting different types of CHD, enabling healthcare providers to make more informed decisions. In addition, it is necessary to conduct additional research to investigate the predictive significance of pulse oximetry by analyzing the relationship between initial measurements and long-term outcomes, including survival rates, surgical procedures, and overall health conditions. Utilizing multivariate analysis to identify maternal and neonatal risk factors would enhance screening protocols, leading to improved detection rates and decreased occurrence of false results. Combining pulse oximetry with other diagnostic techniques can improve diagnoses' accuracy, leading to better patient outcomes.

Furthermore, this research has the potential to provide valuable insights for clinical guidelines and establish a uniform approach to healthcare across different institutions. This would ensure pulse oximetry is used effectively and appropriately in various clinical settings. Examining the suitability of pulse oximetry in various populations, such as healthy full-term infants or those in non-specialized care settings, would help fill in knowledge gaps and evaluate how well current findings can be applied to different situations.

The main limitations of our study are represented by a sample size drawn from a single NICU, which may not be representative of the broader neonatal population, along with the short follow-up period leading to a limited understanding of the long-term outcomes. Our cohort consisted mainly of patients admitted to the NICU, which might not reflect the general neonatal population or those in non-tertiary settings. Additionally, without linking screening outcomes to specific medical interventions, the study overlooks evaluating the effectiveness of post-diagnosis treatment. There are also other limitations to consider, such as the short follow-up period and lack of post-diagnosis treatment effectiveness evaluation. Future studies should address these limitations to enhance the validity and applicability of the research findings.

## Conclusions

Even though CHD can be diagnosed before birth, some defects are harder to detect than others. Therefore, screening methods such as pulse oximetry may help identify critical congenital heart defects before signs are evident. This study evaluated neonatal screening for CHD in a NICU using pulse oximetry and clinical evaluation. It revealed excellent diagnostic accuracy. Future multi-center trials with diverse populations and longer follow-ups are needed to confirm the benefits of early CHD detection and improve outcomes. Pulse oximetry remains a very reliable screening method for CHD. It is accessible, affordable, and available in most hospitals.
